# Mechanical Forces Accelerate Collagen Digestion by Bacterial Collagenase in Lung Tissue Strips

**DOI:** 10.3389/fphys.2016.00287

**Published:** 2016-07-12

**Authors:** Eunice Yi, Susumu Sato, Ayuko Takahashi, Harikrishnan Parameswaran, Todd A. Blute, Erzsébet Bartolák-Suki, Béla Suki

**Affiliations:** Cell and Tissue Mechanics, Department of Biomedical Engineering, Boston UniversityBoston, MA, USA

**Keywords:** cleavage, stiffness, stretch, computational model, network

## Abstract

Most tissues in the body are under mechanical tension, and while enzymes mediate many cellular and extracellular processes, the effects of mechanical forces on enzyme reactions in the native extracellular matrix (ECM) are not fully understood. We hypothesized that physiological levels of mechanical forces are capable of modifying the activity of collagenase, a key remodeling enzyme of the ECM. To test this, lung tissue Young's modulus and a nonlinearity index characterizing the shape of the stress-strain curve were measured in the presence of bacterial collagenase under static uniaxial strain of 0, 20, 40, and 80%, as well as during cyclic mechanical loading with strain amplitudes of ±10 or ±20% superimposed on 40% static strain, and frequencies of 0.1 or 1 Hz. Confocal and electron microscopy was used to determine and quantify changes in ECM structure. Generally, mechanical loading increased the effects of enzyme activity characterized by an irreversible decline in stiffness and tissue deterioration seen on both confocal and electron microscopic images. However, a static strain of 20% provided protection against digestion compared to both higher and lower strains. The decline in stiffness during digestion positively correlated with the increase in equivalent alveolar diameters and negatively correlated with the nonlinearity index. These results suggest that the decline in stiffness results from rupture of collagen followed by load transfer and subsequent rupture of alveolar walls. This study may provide new understanding of the role of collagen degradation in general tissue remodeling and disease progression.

## Introduction

Lung tissue is constantly under the influence of a static preexisting tensile stress, called prestress, due to transpulmonary pressure as well as dynamic stresses imposed by tidal breathing (Suki et al., 2011). The corresponding mechanical forces within the intact tissue influence a variety of normal cell functions including cellular signaling and tissue remodeling (Ingber, 2006). The mechanical stresses are transferred through the ECM via the key load bearing components, elastin and collagen, which play essential roles in defining the mechanical properties of the lungs (Suki et al., 2005).

The development, maintenance and remodeling of the ECM require enzymes such as elastase and collagenase. In normal lung tissue *ex vivo*, the activity of elastase is enhanced by mechanical forces due to unfolding of binding sites and directly increasing the cleaving off rate of elastase (Jesudason et al., 2010). In diseases such as emphysema, mechanical forces can enhance the destruction of tissue structure by rupturing the alveolar walls which are enzymatically weakened by the progression of the disease (Kononov et al., 2001). However, rupture of an alveolar wall is not possible without collagen remodeling.

Since collagen is an integral part of determining the mechanical properties of lung tissue (Suki et al., 2005), the remodeling and destruction of collagen is expected to result in significant changes in the macroscopic mechanical function of the tissue. Previous studies have shown that significant collagen remodeling occurs in diseases, such as emphysema (Finlay et al., 1996; Shapiro and Senior, 1999). One such study used a transgenic mouse line with lung-specific expression of matrix metalloproteinase (MMP)-1 to show that emphysema could develop through an elastin-independent mechanism (D'Armiento et al., 1992). MMPs are proteases that can degrade various components of the ECM (Elkington and Friedland, 2006). MMP-1, specifically, is an interstitial collagenase that degrades the fibrillar collagen types I, II, and III.

A series of studies done by the Ruberti group have demonstrated that the presence of static mechanical loading actually protects collagen from digestion by collagenase (Ruberti and Hallab, 2005; Bhole et al., 2009; Flynn et al., 2010; Zareian et al., 2010; Camp et al., 2011). Most of these studies used wide spectrum bacterial collagenase as well as MMP-8. Accordingly, we formulated the null hypothesis that both static and dynamic stretch on the collagen in the native ECM of the lung parenchyma protect against enzymatic digestion. To this end, we measured the rate of decay of tissue stiffness in the presence of bacterial collagenase during various stretch patterns. The effect of stretch on tissue degradation during digestion was then examined at the micro- and ultra-structural levels using fluorescent and electron microscopy, respectively. The results were interpreted using a network model of the lung parenchyma.

## Methods

### Tissue preparation

All procedures were approved by the Animal Care and Use Committee of Boston University. A total of 58 healthy male C57BL/6 mice weighing 24–26 grams were anesthetized with injection of 70 mg/kg of pentobarbital sodium. Following anesthesia, a tracheotomy was performed, and a cannula was inserted into the trachea. The heart and lungs of the mice were exposed through opening of the chest, and the mice were exsanguinated. The lungs were then perfused with phosphate buffered saline (PBS) through the right ventricle to clear the lungs from blood. The lungs were then excised and sliced into strips with approximate dimensions of 5 × 1 × 1 mm (length × width × thickness). Lung tissue strips (3–4 per lung) were stored in chilled PBS and used within 4–5 h of excision.

### Stretching apparatus

In each tissue strip, stress-strain data were obtained using a previously developed uniaxial tissue stretching system (Araujo et al., 2011). The system consists of a computer-controlled dual-mode lever arm-force transducer system (model 300B, Aurora Scientific, Ontario, Canada) and a separate bidirectional, inductive type force transducer (model LC-01, CSM Instruments, Switzerland). Both instruments are attached to an acrylic test stand housing a 22 mL tissue bath. The entire apparatus was placed on a hot plate and the tissue bath temperature was kept at 37°C. The dual-mode system (total length range of 10 mm, maximum force of 0.5 N) was used to stretch the tissue samples with prescribed displacement-time profiles, and the CSM Instruments force transducer (maximum force of 0.1 N) was used to measure the stress-strain curves. A custom labVIEW program (National Instruments, Austin, TX) was used to operate the stretching system and record displacement and force data. The user-defined displacement signals generated by the computer program were passed through a digital to analog converter, low-pass-filtered at 10 Hz (901P Filter Bank, Frequency Devices, Haverhill, MA), and sent to the lever arm. The recorded force response signals were low-pass-filtered at 10 Hz (901P Filter Bank, Frequency Devices, Haverhill, MA) and sampled by the data acquisition board (DAQCard-6062E, Nation Instruments) and connector block (BNC-2110, National Instruments) at a sampling frequency of 30 Hz.

### Tissue stretching protocol

The bath was filled with 22 mL of Krebs–Ringer Bicarbonate Buffer with 1% bovine serum albumin (Sigma–Aldrich). The ends of the lung tissue strips were attached to small metal plates using cyanoacrylate glue, and the plates were then attached to the lever arm and transducer arm via stainless steel wires. The tissue sample was kept submerged throughout the remainder of the experiment. The tissue sample was preconditioned by applying three, consecutive triangular displacement wave signals, from 0 to 40% strain. After preconditioning, the tissue was allowed to equilibrate for 5 min prior to the start of the experiment. Depending on the specific condition applied (one per strip), bacterial collagenase (Sigma–Aldrich, collagenase from clostridium histolyticum) was added to the bath (6 μg/ml), and mechanical loading was applied. The reference length was the longest length at which the sample did not produce force. Force-length measurements between 0 and 40% strain were taken at regular intervals between periods of continuous mechanical loading over the 1-h testing period.

### Confocal microscopy

A laser scanning confocal microscope (Olympus FV-1000) was used to image the lung tissue structure following the 1-h testing protocol. Samples evaluated included all static conditions, undigested (0, 40, 80% stretch), and digested (0, 20, 40, 80% stretch), and the undigested and digested cyclic 20% amplitude, 1 Hz conditions superimposed on 40% static strain. Since collagen and elastin are autofluorescent in the green spectrum (500–600 nm), no specific labeling was necessary. A 488 nm argon laser excited this emission spectrum. To image throughout the tissue thickness, the confocal pinhole and *z*-axis step size were matched with the optical objective, optimizing the optical sectioning of the lung tissue. All tissue strips were imaged in the unstretched state.

### Electron microscopy

Formalin-fixed tissue strips were additionally fixed in 2.5% glutaraldehyde in 0.1 M phosphate buffer (PB, pH 7.4) for 1–2 h at room temperature. Strips were washed in PB for 3 changes of 10 min each. Samples were post-fixed in 1% osmium tetroxide in PB for 1 h on ice, followed by a final set of washes in PB. Tissue strips were routinely dehydrated with increasing ethyl alcohol concentrations (25, 50, 75, 90, and 100%) using 2 washes of 10 min each. There was an additional 10 min wash in 100% ethyl alcohol prior to the 2 washes of 10 min each in the transition solvent, propylene oxide. Following dehydration, tissue samples were infiltrated with embedding resin, Poly/Bed 812 Embedding Media/DMP-30 Kit (Polysciences, Inc, Warrington, PA). Tissue samples were placed in a 1:2 resin to propylene oxide solution overnight, then in a 2:1 resin to propylene oxide solution, again overnight. Tissues were then placed in 100% resin for 30 min, and finally embedded in flat molds with fresh resin.

Embedded tissue samples were polymerized in a 60°C oven for at least 24 h, cooled, and removed from the molds. The polymerized samples were sectioned for thin tissue samples (between 90 and 200 nm), using an ultra-microtome (Leica Microsystems UltraCut UCT). Sectioned samples were collected, placed on grids, and allowed to dry overnight. Samples were then stained using 2% uranyl acetate in ultrapure water and Reynold's lead citrate.

A transmission electron microscope (Jeol JEM 2010) was used to image the lung tissue. Sections were observed at 80 kV accelerating voltage. Images were captured with Kodak 4489 negatives, which were developed and then scanned using a high-resolution scanner (Agfa DuoScan 2500).

### Data analysis

First, the force and length recordings were converted to stress and strain by dividing force and extension with the initial cross-sectional area and the initial length of the tissue strip, respectively. Stress-strain curves were constructed and analyzed to obtain Young's modulus (Y) values at 15 and 36% strains. To measure the change in modulus, a fourth-order polynomial was fit to the stress-strain curves and the slope of the polynomial at 15% strain was used as Y. The modulus values were obtained at each time point for each test condition and normalized with the value of Y at time 0. To characterize the shape of the stress-strain curve, a nonlinearity index was defined as the relative difference in modulus between 15 and 36% strains: k = (Y(36%) − Y(15%))/Y(15%).

To determine changes in lung tissue structure following digestion and stretch, differences in alveolar morphology were quantified using ImageJ by calculating the equivalent diameter and distortion index of individual alveoli. The equivalent diameter was measured by manually outlining the alveolar walls of airspaces. An ellipse was fit to the outline and the area of the fitted ellipse was obtained. The equivalent diameter of the alveoli was calculated as the diameter of a circle having the same area. The distortion index characterizing the shape of individual alveoli was calculated as the ratio of the major and minor axes (aspect ratio) of the fitted ellipse. Each group included in the analysis contained between 60 and 150 airspaces.

### Network modeling

To better understand how stretch influences digestion, a simple network model simulation was conducted. A hexagonal network consisting of 450 springs was used with a nonlinear force-extension relationship: F = k^*^_0_Δx + k^*^_1_Δx^2^, where k_0_ and k_1_ are the linear and nonlinear spring constants, respectively. The digested, unstretched case was simulated by decreasing k_0_ and k_1_, such that first springs were randomly chosen and the k_0_ and k_1_ of these springs were changed to preset values to mimic the experimental data. The digested and stretched cases were simulated by adding a probabilistic breaking procedure which breaks springs based on local strain given a certain probability, P = P_0_ + c^*^ε, where P_0_ is the probability that a spring will be cut, c is a constant which characterizes the coupling between fiber strain and enzyme activity, and ε is the local strain of the fiber. This is to simulate how, as the collagen is degraded, the loads carried by previously intact fibers are transferred to neighboring fibers post-rupture, which leads to increased strain on these fibers, and a progressive cycle of destruction. We found that for the 80% strain condition, an increased value of P_0_ had to be used, simulating the enhanced enzymatic activity due to the increased stretch. This may correspond to the increase in unfolding binding sites along the fibers similar to what has been reported for lung elastin (Jesudason et al., 2010).

### Statistical analysis

Statistical analyses of the modulus and nonlinearity index across different test groups were done using one- or three-way ANOVA, depending on the comparisons being made. Data were also tested for normality and if the normality test failed the log transformation was used before applying the statistical test or the proper non-parametric test was used. Confidence intervals for medians and variances were predicted using bootstrap method. Statistical significance was defined as *p* < 0.05 for all methods.

## Results

**Figure 1:**
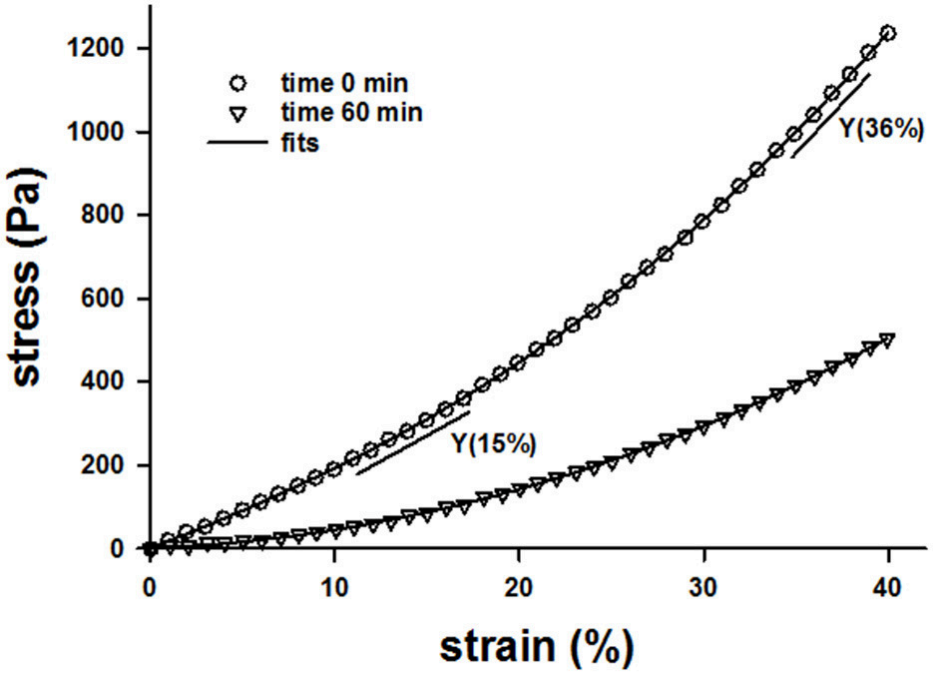
**Example stress-strain curves of a lung tissue strip before digestion at time 0 (circles) and after 60 min of digestion with bacterial collagenase (triangles) while the sample was help at a uniaxial strain of 80%**. The solid lines through the symbols are the fit of a 4th order polynomial and the straight shorter lines are the slopes obtained from the polynomial fits at 15 and 36% strains.

**Figure 2:**
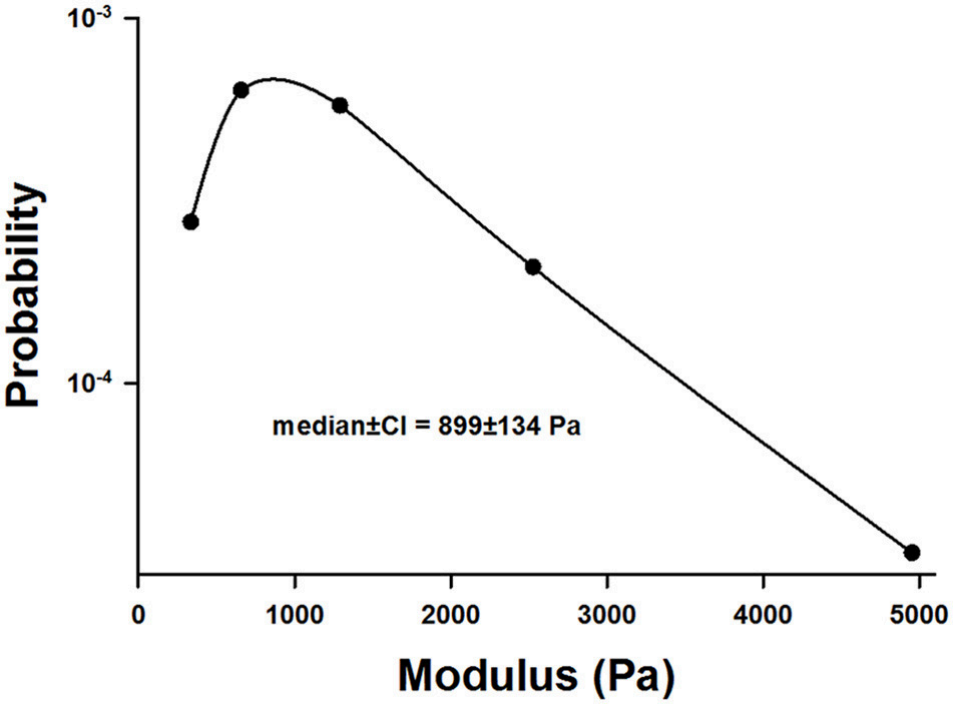
**Distribution of the incremental modulus Y of normal lung tissue strips**. Note the highly asymmetric nature of the distribution.

Example stress-strain curves before and after 60 min of digestion are shown in Figure 1. Both the stress and the Young's modulus (Y), computed as the slope of the curve at 15% strain, are significantly reduced following digestion. Since Y values were obtained for all samples including additional control groups not reported, we obtained a large number of control measurements (*n* = 140) which allowed us to construct a distribution of Y (Figure 2). The distribution is skewed showing an exponential tail with a median and 95% confidence interval of 899 ± 134 Pa.

**Figure 3:**
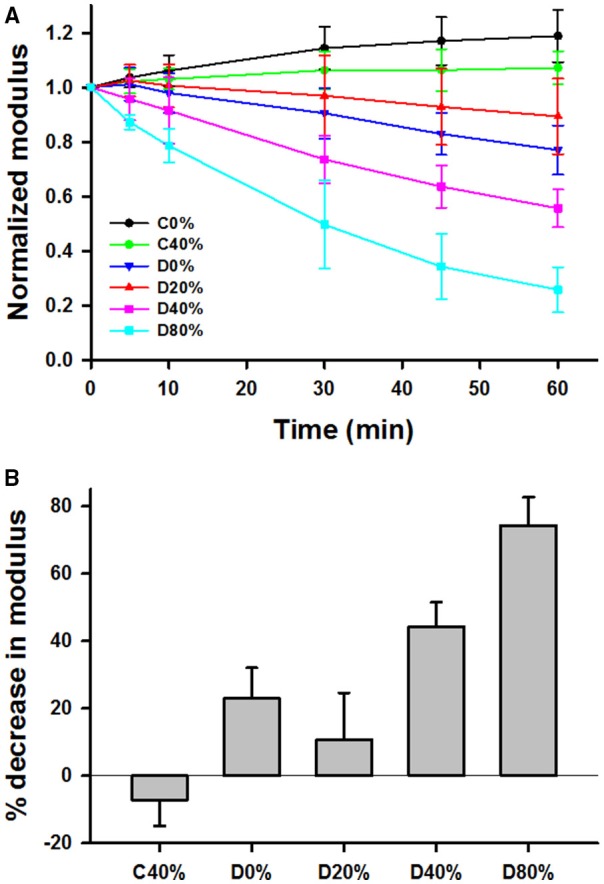
**(A)** Time course of mean and SD of modulus at 15% strain normalized to unity at time 0 during 60 min in the tissue bath with or without adding bacterial collagenase. C0% and C40% are control groups without collagenase held, respectively, at 0 or 40% uniaxial strain during the 60 min. D0%, D20%, D40%, and D80% represent groups that were digested with collagenase and held at 0, 20, 40, or 80% strain, respectively. **(B)** Comparison of the percent decrease in the normalized moduli from time 0 to 60 min. Each bar is statistically significantly different from the other.

The time evolution of Y was normalized to unity at time *t* = 0 for various unstretched and static stretched groups including control unstretched (C0%, *n* = 9), control 40% stretched (C40%, *n* = 7), control 80% stretched (C80%, *n* = 6), digested unstretched (D0%, *n* = 10), digested and stretched at 20% (D20%, *n* = 7), 40% (D40%, *n* = 10), and 80% (D80%, *n* = 7). Generally, the digestion decreased Y in time whereas Y stayed constant (C40%, C80%) or slightly but statistically significantly increased (C0%) in time in the control samples. Since the C80% group was identical to the C40%, it was not included in further analysis. The time courses of Y in the remaining groups are compared in Figure 3A. First, a three-way ANOVA was applied including data from the C0%, C40%, D0%, and D40% groups. This analysis indicated the presence of strong interactions among time, stretch, and digestion. Specifically, we found that (1) the effect of time significantly depended on whether the samples were stretched (40% strain) or not (0% strain; *p* < 0.001), (2) the effect of time also significantly depended on whether the samples were control (C0% and C40%) or digested (D0% and D40%; *p* < 0.001), and (3) the effect of digestion significantly depended on whether the samples were at 0 or 40% strain. Next, we compared the percent decrease in Y of all groups at 60 min (Figure 3B) using one-way ANOVA which showed that all groups were statistically significantly different from each other (*p* < 0.001). It is notable that the drop in Y after 60 min of digestion in the D20% group was significantly smaller than in both the D0% and D40% groups.

**Figure 4:**
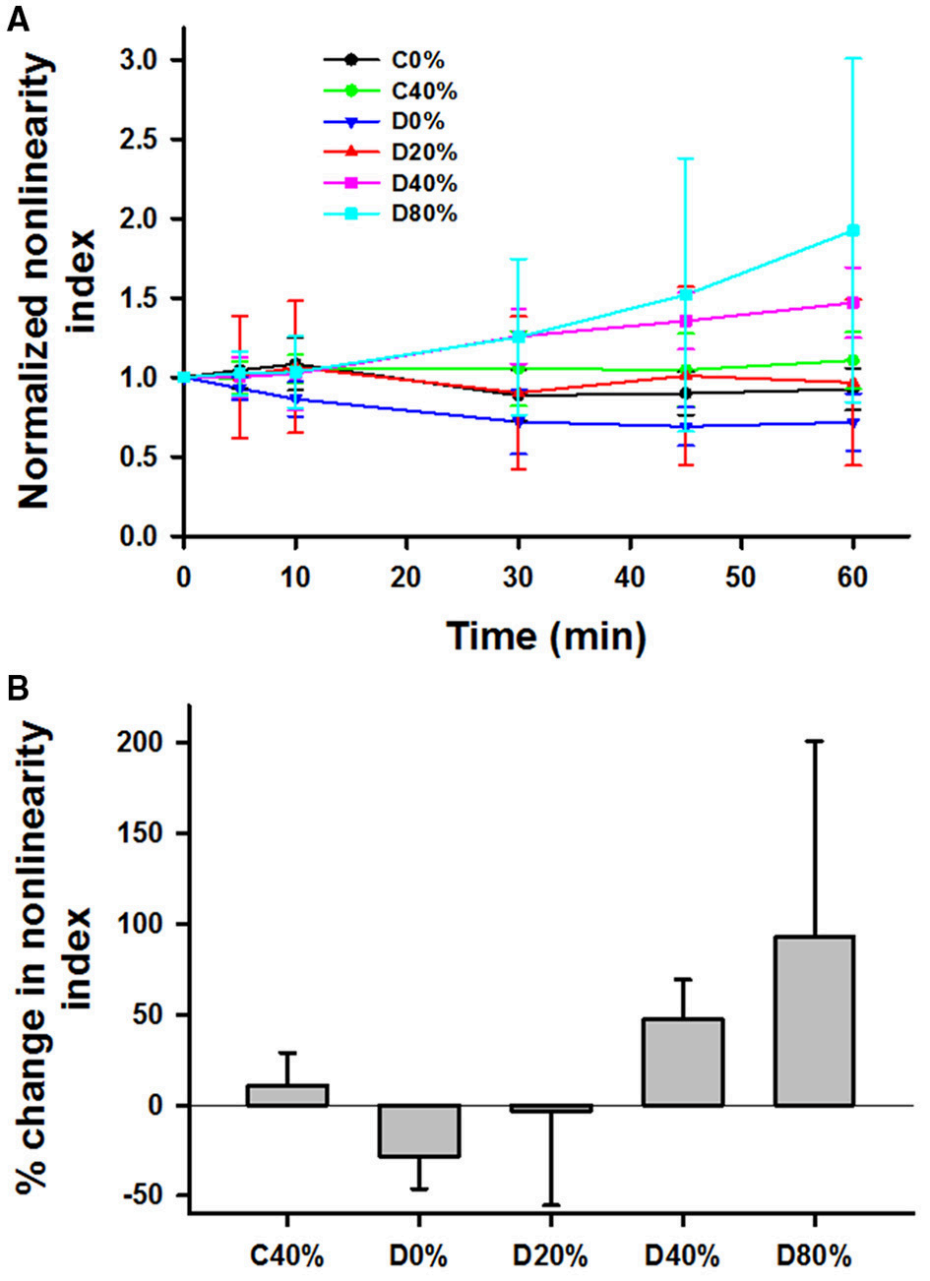
**(A)** Time course of mean and SD of the nonlinearity index defined as the relative change in modulus between 36 and 15% strain, normalized to unity at time 0 during 60 min in the tissue bath with or without adding bacterial collagenase. **(B)** Comparison of the percent increase in the normalized nonlinearity index from time 0 to 60 min. For the definition of the groups see the caption in Figure 3. The D0% group was statistically different from both the D40% and D80% groups.

The nonlinearity index k, normalized to unity at *t* = 0, generally increased with time for the stretched and digested groups (D40% and D80% in Figure 4A). First, limiting the analysis to data at 0 and 40% strains, application of a three-way ANOVA showed that there were statistically significant interactions among time, stretch and digestion (*p* < 0.01). Specifically, the effect of stretch is significant (*p* < 0.001) when averaged over time within the control samples. The effect of time is also significant at 40% strain for the digested samples only (*p* < 0.001). Figure 4B compares the percent increase of k at 60 min using one-way ANOVA. Since the SD values are high, significant difference was only found between the D0% and D40% and the D0% and D80% groups.

**Figure 5:**
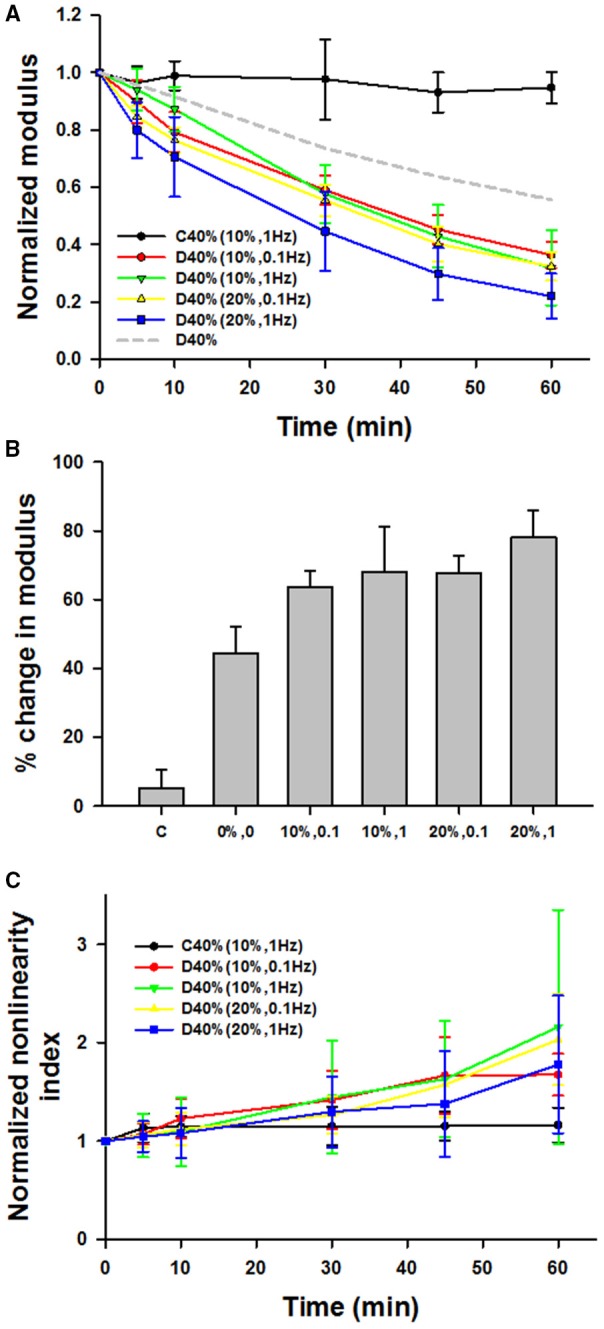
**(A)** Time course of mean and SD of the normalized modulus during 60 min with or without adding bacterial collagenase. The amplitudes and frequencies of sinusoidal stretching around the static strain of 40% are given in parentheses. For comparison, the 40% static stretch data without error bars (D40%) is also shown by dashed gray line. **(B)** Comparison of the percent change in the normalized moduli from time 0 to 60 min. The static D40% (0%0) from Figure 3B is different from all dynamic cases and the D10%–0.1 Hz case different from the D20%–1 Hz case. **(C)** The nonlinearity index for which there was a significant increase with time but there was no difference among the groups.

Figure 5A compares the time courses of the dynamically stretched and digested groups denoted by 10%, 0.1 (*n* = 5), 10%, 1 (*n* = 5), 20%, 0.1 (*n* = 5), and 20%, 1 (*n* = 8) where the first number is the dynamic strain amplitude around the 40% static strain and the second number is the frequency in Hz. Interestingly, all of the dynamic groups showed significantly stronger decrease with time than the static D40% group. Three-way ANOVA demonstrates a significant effect of time (*p* < 0.001) and interactions between frequency and amplitude of stretching around a static mean stretch of 40% (*p* < 0.01). While the effect of time did not depend on what level of frequency or amplitude was present, the effect of frequency (0.1 or 1 Hz) on Y significantly depended on what level of amplitude (10 or 20%) was present (*p* < 0.01). Specifically, a larger decrease was observed (1) at 1 Hz than at 0.1 Hz at 20% dynamic strain amplitude and (2) at 20% amplitude than at 10% amplitude but only at 1 Hz. The percent decreases in Y at 60 min are compared with each other and the static case of D40% (from Figure 3B) in Figure 5B. The static D40% is different from all dynamic cases (*p* < 0.001) and the 10%, 0.1 Hz case is different from the 20%, 1 Hz case suggesting that the higher frequency and larger amplitude cyclic stretching enhances enzymatic degradation of lung tissue. Figure 5C shows the time course of the normalized nonlinearity index for which there was a significant increase with time (*p* < 0.001) but there was no difference among the groups.

**Figure 6:**
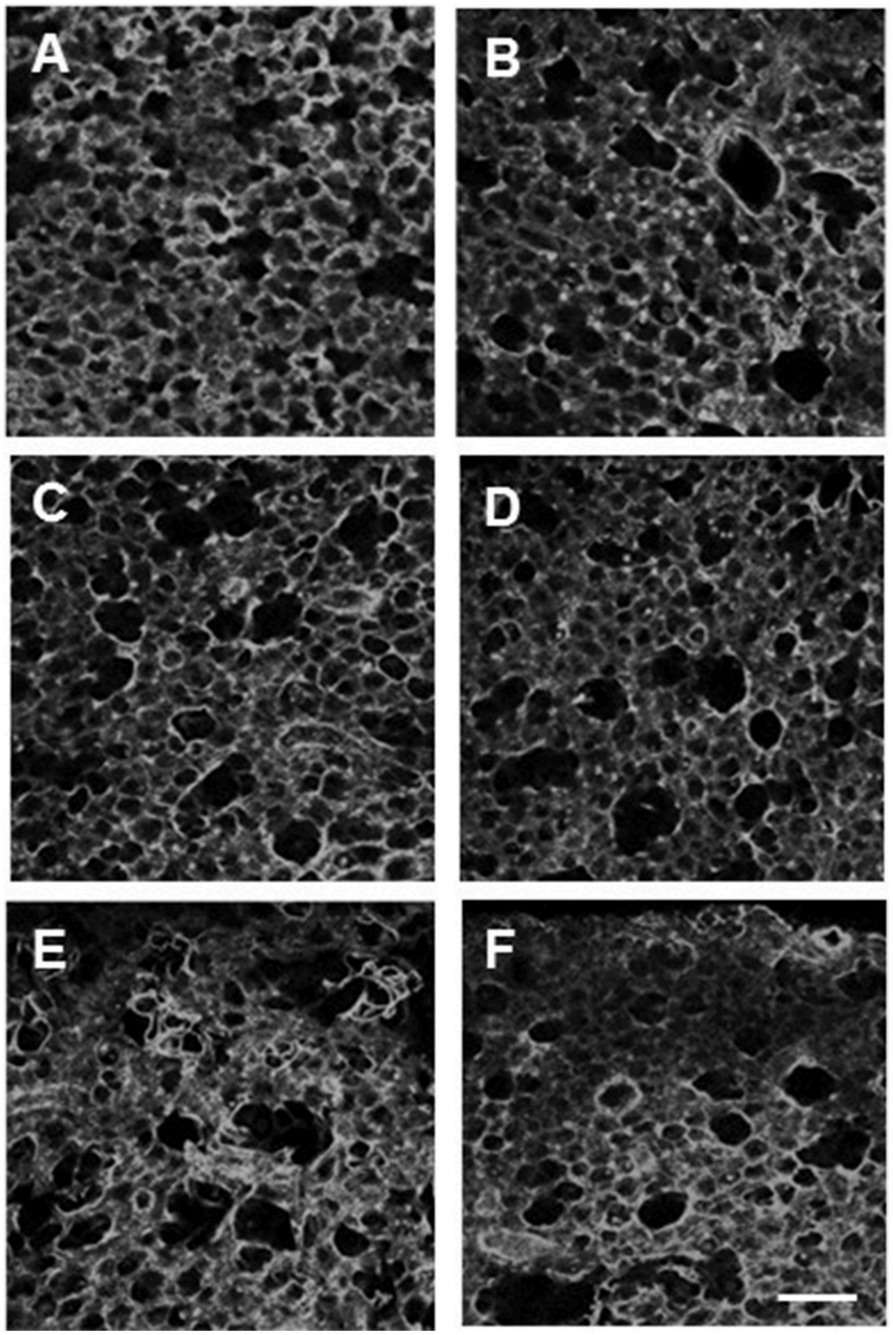
**Examples of confocal images of the alveolar structure in lung tissue strips following 60 min of one of the conditions: no stretch and no digestion (A), no stretch and digestion (B), 40% strain no digestion (C), 40% strain digestion (D), 80% strain and digestion (E), and 20% dynamic strain superimposed on 40% static strain at 1 Hz (F)**. The bar represents 100 μm. In all cases, imaging was done in the unstretched state.

**Figure 7:**
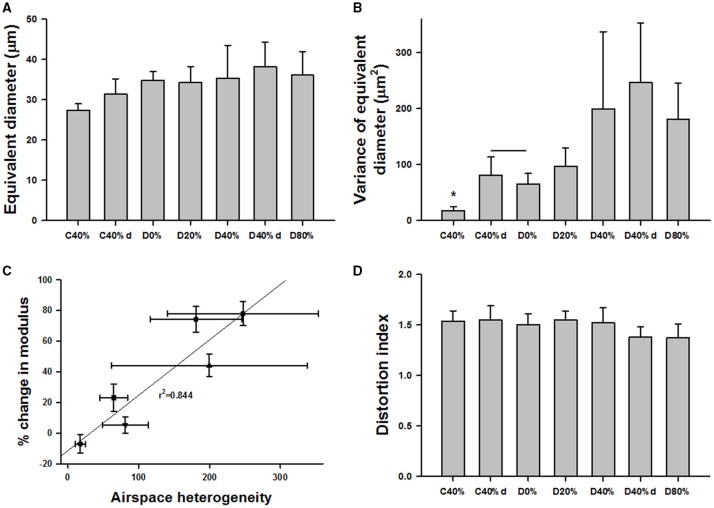
**Structure and function in digested tissue strips. (A)** Equivalent diameters in several control and digested groups. The definitions of the groups are the same as in Figure 3 except that C40%d and D40%d represent dynamic 20% stretch superimposed on 40% static strain at 1 Hz. The diameters in the undigested C40% and C40%d groups were smaller than all digested groups with the exception that D20% was not different from C40%d. **(B)** The variance in C40% was statistically significantly smaller than any other group (^*^). The D40%, D40%d, and D80% groups were not different from each other, but they were higher than the remaining groups. **(C)** The percent change in Y showed a strong correlation with the variance. **(D)** The distortion index was significantly higher in the C40%d group than in the D80% and D40%d groups.

To assess the structural changes in the parenchyma, confocal images of the alveolar wall network were obtained for selected groups including C0%, C40%, C40%d (control samples at 40% static stretch and superimposed dynamic stretch at 20% amplitude at 1 Hz), D0%, D20%, D40%, D80%, and D40%d = 20%,1. Example images are summarized in Figure 6 and statistical analysis of the structure is shown in Figure 7. The equivalent diameters (Figure 7A) in the undigested C40% and C40%d groups were smaller than all digested groups (*p* < 0.001) with the exception that D20% was not different from C40%d. The equivalent diameter of the C40%d group was also statistically significantly higher than that of C40%. We also examined the heterogeneity of the airspace structure by comparing the variance of equivalent diameters in Figure 7B. The heterogeneity was the smallest in the C40% group and this was statistically significantly smaller than the heterogeneity of all other groups. The D40%, D40%d, and D80% groups were not different from each other, but they were higher than the remaining groups. Additionally, the C40%d group showed higher heterogeneity than the D0% group. Interestingly, the percent drop in Y showed a strong correlation with airspace heterogeneity (Figure 7C) with an *r*^2^ of 0.844 (*p* = 0.01). The distortion index (Figure 7D) was higher in the C40%d group than in the D80% and D40%d groups (*p* < 0.001).

**Figure 8:**
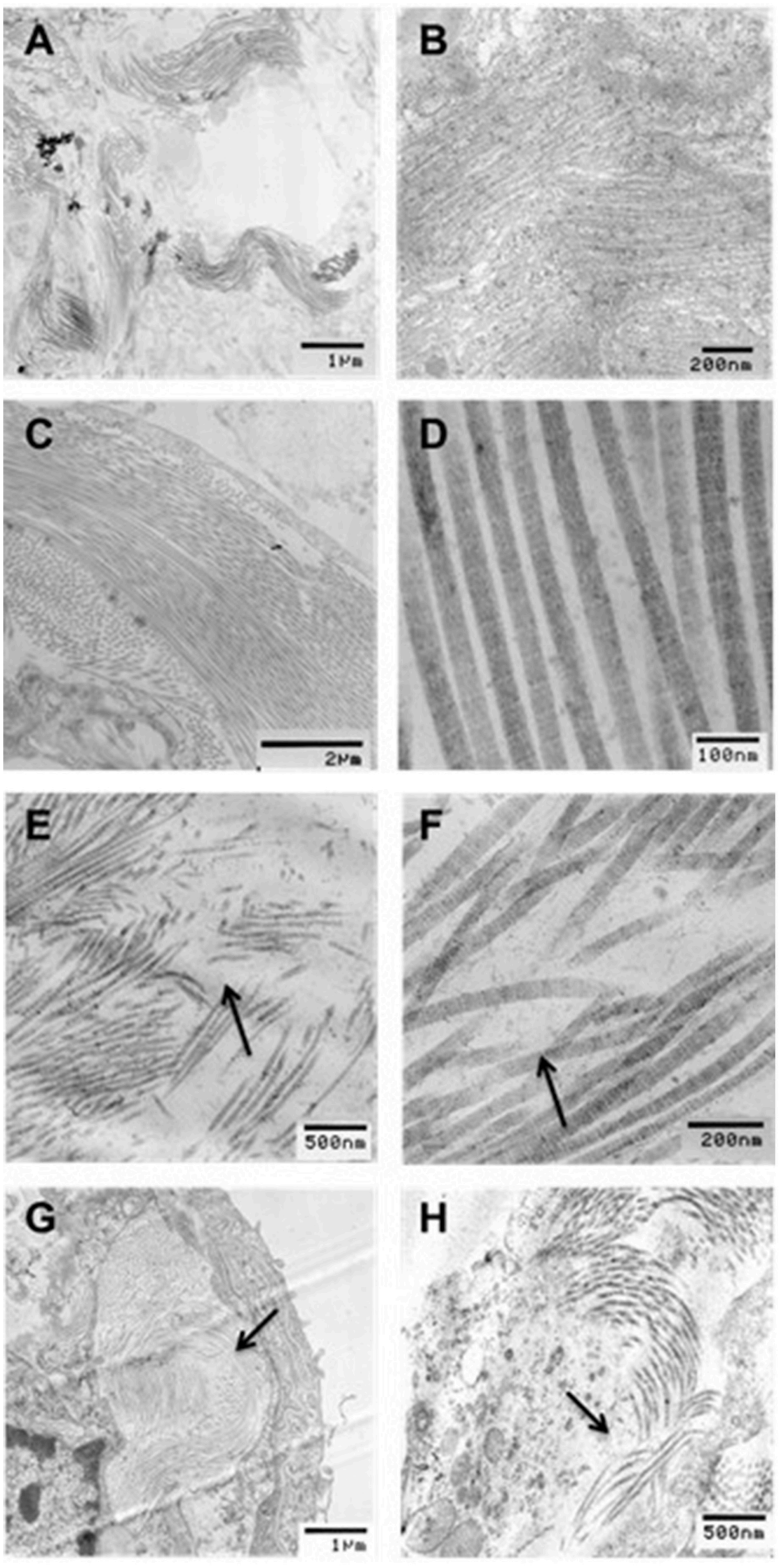
**Transmission electron microscopy (TEM) images of collagen structure for (A,B) undigested, no stretch, (C,D) undigested, 40% strain, (E,F) digested, no stretch, and (G,H) digested, 40% strain**. All images were taken in the unstretched state. Arrows indicate regions of collagen degradation. Bar denote magnification.

To determine the structural changes at the level of collagen fibrils, electron microscopic images were also obtained in selected groups including C0%, C40%, D0%, and D40% (Figure 8). It can be seen that the fibers are wavy and closely packed when no enzyme and stretch was present, but they were straighter after stretch even though imaging was obtained in the unstretched condition. The fiber structure became irregular, loosely packed and damaged in the presence of enzymes. When enzymes were applied in the presence of stretch, the structure became heterogeneous with both intact and highly damaged collagen fibers. Large regions that contained little or no collagen were often seen in the digested and stretched tissues.

**Figure 9:**
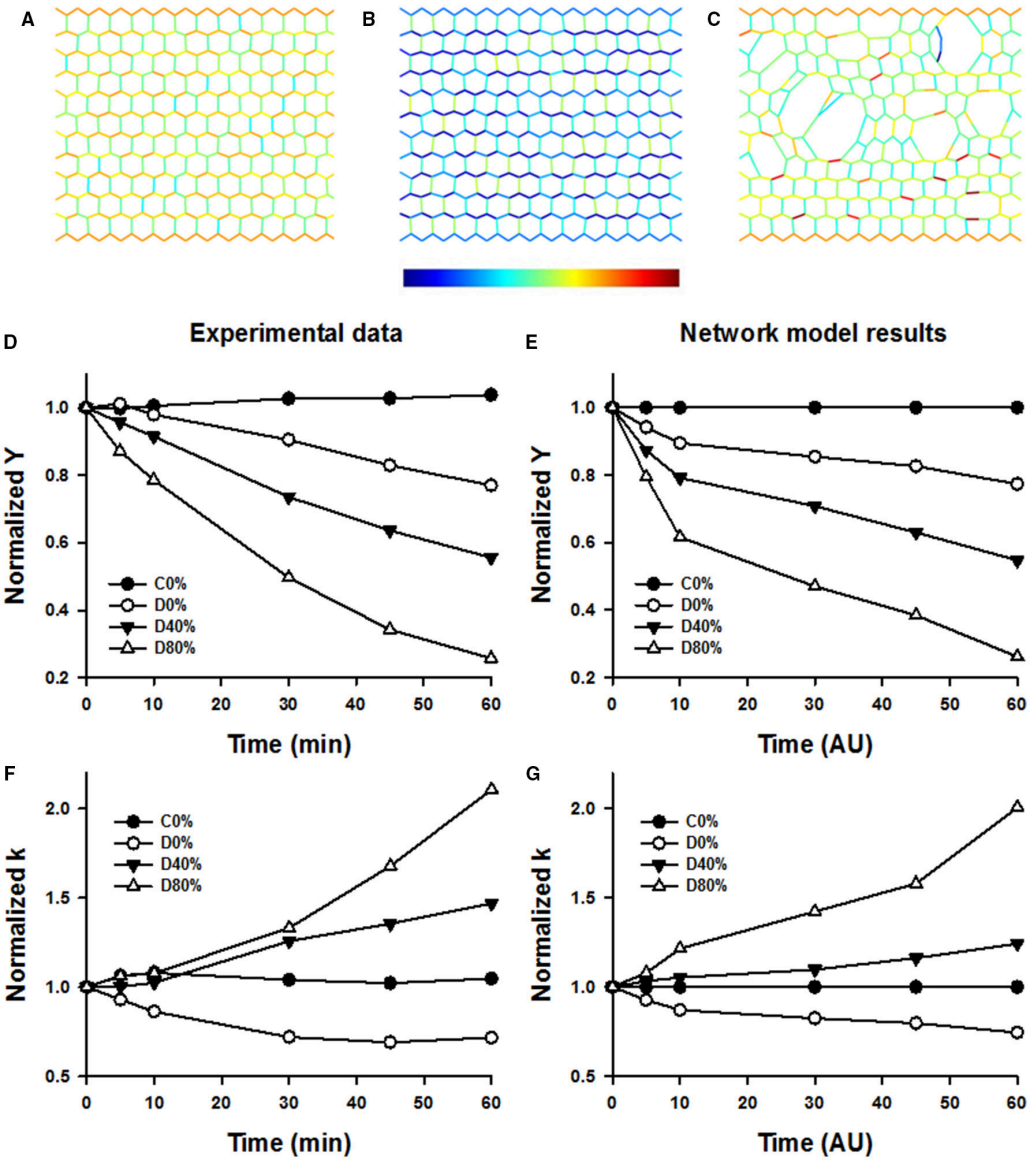
**Top row: Network images under simulated conditions of no enzyme and static 40% strain (A), enzyme and no stretched (B), and enzyme and static 40% strain allowing breaking (C)**. Color is proportional to force (color bar below **B**) increasing from blue to red. Note the increased degradation and heterogeneity in structure in **(C)**. Note also that in **(B)** colors are bluish representing less force due to weakening of springs whereas in **(C)** colors are similar to those in **(A)** despite weakening of springs. This is due to load transfer following breaking. **(D,F)** shows selected time courses of experimentally obtained normalized Young's moduli (Y) and normalized nonlinearity index k. **(E,G)** shows normalized Y and k from the network model demonstrating a good correspondence with the experimental data.

Figure 9 shows example configurations of the network model before (Figure 9A) and after digestion in the absence (Figure 9B) and presence (Figure 9C) of stretch. The stiffness of the networks was calculated at 15 and 36% strains along the stress-strain curve similarly to the experimental data. The normalized stiffness and nonlinearity index are compared to the corresponding experimental results in Figures 9D–G. It can be seen that the network model matches the trends seen in the experimental data well. The network image in Figure 9C also displays a similar pattern of airspace enlargement seen in the confocal images in Figure 6.

## Discussion

In this study, the baseline variability of the measurements was high with the stiffness covering an order of magnitude from about 500 to 5000 Pa (Figure 2). To explain this, note that stress was defined as the force divided by the undeformed cross-sectional area which was obtained by measuring the width and thickness of the strip prior to the start of the experiment. However, the accuracy of such measurements was hindered by the difficulty of slicing fresh strips to a uniform width and thickness. This uncertainty likely introduced inter-sample variability both in stress and Y. In addition, stretching a sample causes the thickness and width to decrease with increasing strain, due to the Poisson effect. Thus, uneven slicing can also contribute inter-sample variability via tissue nonlinearity and the Poisson effect. Nevertheless, the most important source of the variability in Y is likely due to the varying amounts of airway segments in different samples (Salerno et al., 1995).

A limitation of this study is the use of bacterial collagenase rather than a more physiologically relevant enzyme such as MMP-1 which has been shown to be present in increased concentrations in humans with late-stage emphysema (Imai et al., 2001). The difference between the two collagenases lies in the specificity of the enzyme. Bacterial collagenase is much less restrictive in its activity, and is capable of degrading both native and denatured collagen at multiple sites into smaller peptides (French et al., 1987). On the other hand, tissue collagenases, such as MMP-1, are highly selective and only degrade native collagens at a single site (Manka et al., 2012). This difference together with the high dose (6 μg/ml) compared to *in vivo* concentrations of MMP-1 in emphysematous lung (< 22 ng/ml; Imai et al., 2001) may have contributed to an exaggerated effect of collagen degradation. However, studying the mechanism of digestion is still informative and indeed bacterial collagenase has often been used in the past (Huang and Yannas, 1977; Yuan et al., 2000; Ruberti and Hallab, 2005; Bhole et al., 2009; Wyatt et al., 2009; Camp et al., 2011). Additionally, investigating the effects of bacterial collagenase may also have implications for pathologies, such as bacterial infections.

There are additional limitations associated with the confocal microscopy because the images show a 2D plane within the tissue strips. As such, parts of the alveolar walls are not visible. Therefore, choosing which alveoli to analyze is somewhat subjective and the 2D analysis itself brings in significant baseline heterogeneity (Parameswaran et al., 2009). Furthermore, if an alveolus on a 2D section contains its opening to the alveolar duct, the full outline of the alveolus is not clear. To minimize artifacts, only those alveoli were included in the analysis which appeared to be completely within the plane of the image. Since imaging was obtained in the unstretched condition, it was difficult to determine the orientation of stretch once the samples were sectioned and loaded into the microscope. With regard to the digestion results, it is possible that the lateral contraction at higher stretches allowed a deeper penetration of the enzyme into the tissue which may contribute to a larger apparent decrease in Y. On the other hand, the strain is the largest around the boundary of the sample with fixed end dimensions and for elastin, we previously observed that more enzymes bind near the surface which produces a screening effect (Jesudason et al., 2010). Additionally, it may be possible that the larger stretches (e.g., 80%) already damage collagen which would promote accelerated digestion by the enzyme. We believe this is not the case because the C80% group did not show stiffness decline and the failure strain of lung tissue is above 200% (Ito et al., 2005). Finally, a limitation of the modeling is that the network structure is 2D whereas our sample having a cross-sectional thickness of 1 mm, could include at least 20 layers of alveoli based on alveolar dimensions (Osmanagic et al., 2010). However, this is unlikely to influence the modeling conclusions since mechanical force-based tissue destruction in 3D (Parameswaran et al., 2011) and 2D (Jesudason et al., 2010) models provide similar stiffness decreases.

Our results indicate that under most conditions, mechanical forces enhance the irreversible degradation of collagen in lung tissue by bacterial collagenase in accord with findings on elastin (Jesudason et al., 2007, 2010; Chow et al., 2013) and collagen (Ellsmere et al., 1999). While 80% uniaxial strain is outside of the physiological range, this particular strain was tested in response to previous work from the Ruberti laboratory, which showed that applied strain mostly in a lower strain regime (6–10%) had a protective effect on collagen digestion (Ruberti and Hallab, 2005; Bhole et al., 2009; Flynn et al., 2010; Camp et al., 2011). Since the orientation of collagen is random and at low lung volume collagen is also wavy (Suki et al., 2005), it is possible that the collagen fibers in our tissue samples were not stretched completely even at 40% strain. Consequently, we added the 80% group, a strain at which the collagen within the tissue should be completely stretched. However, we observed a greater decline in stiffness at 80% than at 40% static strain (Figure 3B) and at 40% strain dynamic stretching further accelerated the digestion (Figure 5A) in agreement with a previous study in pericardium (Ellsmere et al., 1999). Consequently, we also added a group at 20% strain which confirmed the existence of protective role of strain, These results suggest that static strain in the lung is also protective against bacterial collagenase digestion, but the range over which this applies is limited to somewhere between 0 and 40% uniaxial strains.

It is important to point out some differences between our study and the previous work. In most cases, a purified network or individual fibrils of type I collagen were used in uniaxial tension (Ruberti and Hallab, 2005; Bhole et al., 2009; Flynn et al., 2010; Camp et al., 2011). In another study, similar observations were made in corneal tissue strips confirming the notion that small uniaxial strain on type I collagen is protective against degradation by bacterial collagenase (Zareian et al., 2010). Interestingly, in the latter study, the cleavage rate increased with strain up to 6.5% and flattened thereafter. Similar results were obtained in rat tail tendon (Wyatt et al., 2009). However, in these studies, the collagen fibrils were initially highly aligned with or perpendicular to the direction of loading which is not the case in the lung. Additionally, the proportion of type III to type I collagen is about 0.3 in the normal lung (Nerlich et al., 1987) which may have contributed to the different results in our study. Nevertheless, the lung is never under static stretch and 20% uniaxial strain would represent a very low lung volume (Suki et al., 2011). At 40% uniaxial strain, superimposing dynamic stretch, both at 10 or 20% amplitude and at 0.1 or 1 Hz, enhanced degradation compared to static strain (Figures 5A,B). In diseases such as emphysema, it is possible that certain regions trap air and hence experience a near static stretch. Thus, while the protective mechanism under small static stretch has likely no physiological benefit in the normal lung, it may effect regional tissue degradation in diseases.

At the single molecule level, the application of 10 pN tensional force on collagen peptides increased cleavage rate nearly 100-fold (Adhikari et al., 2011) which qualitatively agrees with our results. In order to reconcile these contradictions, atomistic level molecular dynamics simulations demonstrated that in the absence of tensile force, the heterotrimer of type I collagen including two α1 and one α2 chains is unfolded in its equilibrium state (Chang et al., 2012). Because unfolding leads to a loss of the triple helical structure near the cleavage site, enzymes may have easier access to each α chain which in turn should accelerate the hydrolysis of the bonds. However, in the presence of tensile force, the triple helical structure is stabilized which hinders access of the enzyme to the α chains and this results in a protecting mechanism against enzymatic cleavage. Interestingly, the homotrimer of type I collagen has a stable triple helical structure even in the absence of tensile force that results in slowing down collagen breakdown (Chang et al., 2012). Furthermore, tensile force can unwind the homotrimer molecule and enhance collagen breakdown. While these mechanisms may operate in isolated molecules and fibrils, inside the complex ECM, collagen is also cross-linked that is missing in reconstituted collagen networks (Flynn et al., 2010). Additionally, collagen in the native ECM is also linked to elastin and proteoglycans that may influence degradation. Indeed, it has been shown that proteoglycans can stabilize collagen in tissues *in vivo* (Snowden, 1982). Furthermore, proteoglycans decorate collagen (Fratzl and Daxer, 1993) which may hinder the accessibility of the cleavage sites. Application of mechanical force to lung tissue could alter the spatial arrangement of proteoglycans around the collagen fibrils so that the diffusion of enzymes from one binding site to another may be enhanced with an increased effective cleavage rate in the presence of tissue stretch. Finally, it is also possible that strain on the fibril induces increased availability of binding sites for the enzymes similarly to elastin (Jesudason et al., 2010) again resulting in an enhanced breakdown. The actual mechanism at the molecular level warrants further investigation.

For the dynamically stretched conditions, we observed that increasing both the amplitude and frequency resulted in a greater decline in stiffness (Figures 5A,B). Since increasing the amount of applied static strain between 20 and 80% leads to greater stiffness decline, superimposing larger cyclic amplitude upon the 40% static strain also increases the maximum applied strain. In the 10% amplitude group, the maximum stretch applied is 50%, while in the 20% amplitude group the maximum stretch is 60%. The structure of the parenchyma is also similar during 80% static digestion and 40% static plus 20% dynamic digestion (Figure 6E vs. Figure 6F). Thus, the results obtained from the dynamic conditions follow the same trend as from the static stretch conditions. Increasing the frequency of stretch also led to a greater decline in stiffness. One possible explanation is as follows. Enzymes typically cleave as they unbind. If the unbinding event is promoted by the peak strain, then the reaction cycle is faster at higher stretching rates because peak strains occur more often. Another possibility may arise from the fact that tissues are viscoelastic. A higher stretching rate generates higher viscoelastic stresses inside the tissue, which in turn should transmit larger forces on molecular bonds increasing the probability of the unbinding event. Given that both enzymes and mechanical forces lower the energy barrier for bond dissociation, we speculate that their combined effect may synergistically amplify cleavage as follows. At the single molecule level, Bell proposed that the off rate of a bond exponentially increases with the force on the bond (Bell, 1978). Although bond strength increases, the corresponding survival time of the bond decreases very fast with increasing loading rate (Evans and Calderwood, 2007). Even if the failure strength of bonds increased at the higher frequency, our apparatus provided the necessary mechanical energy to stretch the fibrils and bonds within the fibrils. Thus, our rate dependence of stiffness decline (Figures 5A,B) may be a consequence of lowering bond survival time.

While our results do not reveal mechanisms at the level of a single collagen molecule, examination of the nonlinearity index can reveal mechanisms at the level of the alveolar wall network. The application of strain led to an increase in the nonlinearity index, while pure digestion resulted in a decrease in nonlinearity index (Figures 4, 5C). The nonlinear mechanical properties of the lung tissue (Figure 1) are mostly attributed to the properties of collagen (Suki et al., 2011). The nonlinearity of collagen results from the effect of recruitment (Maksym and Bates, 1997; Maksym et al., 1998; Yuan et al., 1999; Suki and Bates, 2011). When collagen is folded and wavy, the fibrils experience little force at low strains. However, as strain is increased, the collagen begins to straighten as well as fold in the direction of macroscopic strain. As a result, more and more fibrils and fibers become stretched and the effective stiffness of the tissue increases. Therefore, the extent to which the collagen fibers become stretched determines the tissue nonlinearity. In our experiments, increasing strain results in a decreased stiffness but an increased nonlinearity index. A possible explanation for this is that as collagen is degraded during the testing period, the loads that were being carried by the previously intact collagen fibers are transferred to neighboring fibers post-rupture. In this manner, these fibers experience a greater amount of force, and more fibers become recruited in the direction of strain. This in turn leads to greater tissue nonlinearity, whereas due to rupture, fewer fibers contribute to the stress-strain curve and the global stiffness decreases. The computational modeling results (Figure 9) fully support this interpretation.

The mechanism of load transfer is important and has been shown to contribute to the progression of emphysema (Suki et al., 2003; Ito et al., 2005). The imaging results in Figures 6, 7 also directly support this mechanism. Since increasing applied strain enhanced the decline of tissue function due to degradation of collagen, we expected to see a greater number of ruptured alveolar walls at higher strain. This notion is strengthened by the high correlation (0.87) between stiffness decline and alveolar diameter (not shown). We also found greater structural heterogeneity characterized by the variance of diameters at higher strains (Figure 7B). Interestingly, the decline in stiffness also showed a significant and strong correlation with structural heterogeneity (Figure 7C) implying that mechanical failure at the level of alveolar walls plays a crucial role in functional decline. Surprisingly, however, the distortion index measurements showed that increasing strain in the presence of digestion resulted in less distortion of the alveoli. This indicates that the alveoli are less elongated and more circular in shape. This phenomenon can also be explained with the mechanism of load transfer. As the tissue is stretched, the alveolar walls parallel to the direction of stretch experience a greater amount of mechanical force. This directional increase in load leads to preferential digestion of the collagen in walls carrying higher force leading preferential rupture of alveolar walls. Following rupture, the resulting airspace becomes more circular, as neighboring alveoli coalesce. Indeed, the simulation results suggest the formation of globular airspace enlargement during degradation of the network as can be seen in Figure 9C.

In conclusion, we have shown that applied mechanical load generally leads to a greater decline in stiffness during collagen digestion in lung tissue strips. In the case of 20% static strain, however, the stiffness decreased less than during unstretched digestion, indicating that with the application of a small amount of strain, the resistance of collagen to degradation is higher. Increasing applied strain also leads to an increase in nonlinearity of the stress-strain curve, suggesting a mechanism of load transfer that results in collagen fibers taking on higher forces due to the failure of neighboring fibers. Furthermore, increasing the amplitude and frequency of cyclic mechanical loading enhances stiffness decline. These results indicate that mechanical forces regulate enzymatic activity both at the functional and structural levels.

## Author contributions

EY Designed and carried out mechanics and imaging experiments, analyzed data, wrote manuscript. SS carried out mechanics experiments and analyzed data. AT carried out mechanics experiments and analyzed data. HP analyzed data. TB designed and helped with electron microscopy. EB designed experiment analyzed data. BS designed experiments, analyzed data, and wrote manuscript.

### Conflict of interest statement

The authors declare that the research was conducted in the absence of any commercial or financial relationships that could be construed as a potential conflict of interest.
